# Smooth muscle dysfunction in the pre-inflammation site in stenotic Crohn’s-like colitis: implication of mechanical stress in bowel dysfunction in gut inflammation

**DOI:** 10.3389/fphys.2023.1215900

**Published:** 2023-07-14

**Authors:** John C. Johnson, Ramasatyaveni Geesala, Ke Zhang, You-Min Lin, Amosy E. M’Koma, Xuan-Zheng Shi

**Affiliations:** ^1^ Department of Internal Medicine, University of Texas Medical Branch, Galveston, TX, United States; ^2^ John Sealy School of Medicine Class of 2025, University of Texas Medical Branch, Galveston, TX, United States; ^3^ Department of Pathology, University of Texas Medical Branch, Galveston, TX, United States; ^4^ Department of Biochemistry, Cancer Biology, Neuroscience and Pharmacology, Meharry Medical College School of Medicine, Nashville, TN, United States

**Keywords:** mechanical stress, stenosis, Crohn’s disease, cyclooxygenase-2, motility, smooth muscle

## Abstract

**Background and Aims:** Gut smooth muscle dysfunctions contribute to symptoms such as abdominal cramping, diarrhea, and constipation in inflammatory bowel disease (IBD). The mechanisms for muscle dysfunctions are incompletely understood. We tested the hypothesis that mechanical stress plays a role in muscle dysfunction in a rat model of Crohn’s-like colitis where inflammatory stenosis leads to mechanical distention in the pre-inflammation site.

**Methods:** Crohn’s-like colitis was induced by intracolonic instillation of TNBS (65 mg/kg) in Sprague-Dawley rats. Control rats were instilled with saline. The rats were fed with either regular solid food or exclusively liquid diet. Rats were euthanized by day 7.

**Results:** When rats were fed with solid food, TNBS treatment induced localized transmural inflammation with stenosis in the instillation site and marked distention with no inflammation in the pre-inflammation site of the colon. Smooth muscle contractility was suppressed, and expression of cyclo-oxygenase-2 (COX-2) and production of prostaglandin E_2_ (PGE_2_) were increased not only in the inflammation site but also in the pre-inflammation site. Liquid diet treatment, mimicking exclusive enteral nutrition, completely released mechanical distention, eliminated COX-2 expression and PGE_2_ production, and improved smooth muscle contractility especially in the pre-inflammation site. When rats were administered with COX-2 inhibitor NS-398 (5 mg/kg, i. p. daily), smooth muscle contractility was restored in the pre-inflammation site and significantly improved in the inflammation site.

**Conclusion:** Colonic smooth muscle contractility is significantly impaired in stenotic Crohn’s-like colitis rats not only in the inflammation site, but in the distended pre-inflammation site. Mechanical stress-induced expression of COX-2 plays a critical role in smooth muscle dysfunction in the pre-inflammation site in Crohn’s-like colitis rats.

## Introduction

Inflammatory bowel disease (IBD), including Crohn’s disease (CD) and ulcerative colitis (UC), represents a group of chronic gut inflammatory disorders with remitting and relapsing features that lead to significant disruption in bowel function and patient quality of life (Hendrickson et al., 2002; Fakhoury et al., 2014; Ramos and Papadakis, 2019; Park et al., 2020). IBD presents a significant burden on Western countries with approximately 1.6–1.8 million Americans and over 2 million Europeans currently living with the condition. The numbers are rising in both developed and developing countries (Nguyen et al., 2014; Kaplan, 2015; Ramos and Papadakis, 2019). Additionally, patients living with IBD over a 10-year period incur a nearly 3-fold higher annual mean healthcare costs than people not living with IBD (Park et al., 2020), primarily due to frequent hospitalizations, emergency department visits, expensive medication treatment, and surgical costs.

Patients with IBD suffer from symptoms such as abdominal cramping, diarrhea, and rectal bleeding during active inflammation or relapses. Previous investigations have shown that gut smooth muscle dysfunctions account for cramping, diarrhea, and constipation (Shi et al., 2003; Sarna et al., 2006; Lomax et al., 2017). Those studies focused on the bowel site with inflammation and suggested that immune cells-derived cytokines and inflammatory mediators might contribute to smooth muscle dysfunction (Shi et al., 2003; Sarna et al., 2006; Lomax et al., 2017). However, treatments to reduce inflammation may not necessarily improve bowel function (Snape et al., 1991; Annese et al., 1997; Bickelhaupt et al., 2013; Menys et al., 2016). Moreover, recent studies found that bowel segments other than the inflamed site showed aberrant motility in IBD patients (Bickelhaupt et al., 2013; Menys et al., 2016). These observations indicate that inflammation-independent mechanisms may be involved in the development of smooth muscle dysfunctions and bowel symptoms in IBD patients.

The gastrointestinal (GI) tract is subjected constantly to mechanical stress as intraluminal contents (gas, food, liquid) pass through it (Shi, 2017; Geesala et al., 2021). During an inflammatory state, especially in transmural inflammation such as CD, inflammatory infiltrates, fibrosis, edema, and tissue deformation can significantly increase mechanical stress to the bowel wall (Shi, 2017; Geesala et al., 2021). More importantly, inflammation or fibrosis can cause a narrowing of the bowel wall (stenosis) and create a partial obstruction, resulting in luminal distension (circumferential mechanical stretch) at the site proximal to inflammation (Shi, 2017; Geesala et al., 2021). In fact, one of the most frequently encountered complications of CD is obstruction of the GI tract due to inflammation or fibrosis (Fakhoury et al., 2014; Rieder, 2018; Yoo et al., 2020; Geesala et al., 2021). In the present study, we tested if distention-associated mechanical stress, a non-inflammatory factor, may contribute to smooth muscle dysfunction in a rodent model of stenotic Crohn’s-like colitis induced by intracolonic instillation of TNBS (Geesala et al., 2022). With this model, TNBS induces colonic transmural inflammation with stenosis in the site of instillation and marked distention in the site prior to stenosis (pre-inflammation site) (Geesala et al., 2022).

Cyclooxygenase-2 (COX-2) is an inducible enzyme responsible for converting arachidonic acid to prostaglandins (PG), such as PGE_2_, which mediates inflammatory responses and has profound effects on gut smooth muscle function (Lin et al., 2012). COX-2 is implicated in the pathogenesis of obstruction-induced bowel dysfunctions as mechanical stretch induces potent expression of COX-2 in gut smooth muscle cells (SMC) (Shi et al., 2011a; Li et al., 2012; Lin et al., 2014; Lin et al., 2015). We hypothesize that mechanical stress in the distended site prior to stenosis may induce mechanosensitive expression of COX-2, contributing to smooth muscle dysfunction in the model of stenotic Crohn’s-like colitis. To prove that increased expression of COX-2 in the pre-inflammation site is a mechanical stress-dependent mechanism, we compared the expression levels of COX-2 in the site proximal to stenosis (with mechanical distention) and the site distal to stenosis (with no distention) in the stenotic CD-like colitis model. We also compared the site-specific changes of muscle contractility and expression of COX-2 in colitis when rats were fed with either regular solid food or exclusively liquid diet. Liquid diet treatment, which mimics exclusive enteral nutrition (EEN) in clinical practice (Narula et al., 2018; Ashton et al., 2019), was found to eliminate fecal retention and prevent mechanical distention in the colon (Lin et al., 2021). Furthermore, we determined if COX-2 inhibitor could restore colonic smooth muscle function in the CD model.

## Methods

### Rodent model of Crohn’s-like colitis

All the animal protocols were approved by the Institutional Animal Care and Use Committee of the University of Texas Medical Branch in Galveston, Texas. Experiments were performed in accordance to the Guide for the Care and Use of Laboratory Animals of the National Institutes of Health, United States.

Male and female Sprague-Dawley rats of 8–9 weeks old (200–280 g, Harlan Sprague Dawley, Indianapolis, IN) were used in the study. Rat model of CD-like colitis was induced by intracolonic instillation of TNBS (Sigma Chemical, St. Louis, MO) as described previously (Shi et al., 2011b; Geesala et al., 2022). Prior to TNBS instillation, rats were given bowel cleanser PEG 3350 (GoLYTELY, Braintree, MA) for 24 h and fasted for 16 h. This was to cleanse the colon to minimize the impact of fecal pellets on TNBS absorption in different rats. TNBS (65 mg/kg in 250 µL of 40% ethanol) was instilled in 1 min through an intracolonic catheter to the colon 7 cm from the anus. Control rats were treated with intracolonic instillation of 250 µL saline. Control and TNBS rats were housed separately and fed *ad libitum* with regular pellet foods (Picolab Rodent Diet 5,053, LabDiet, St. Louis, MO) unless specified otherwise. The average daily intake of the pellet food for a control rat is nearly 20 g with ∼70 kcal (4.0 g of protein, 2.0 g of fat, 10.4 g of carbohydrate and 1.0 g of fiber). Rats were euthanized 7 days after saline or TNBS treatments. Body weight was documented over 7-day course. Inflammation was scored 1 to 3 in each rat according to criteria described previously (Shi et al., 2011b).

In the experiments involving *in vivo* inhibition of COX-2 activity, control and TNBS-instilled rats were treated with COX-2 inhibitor NS-398 (Cayman Chemical, Ann Arbor, MI) daily at 5 mg/kg intraperitoneally (ip) in 250 µL of 20% DMSO starting on the day inflammation was induced (Shi et al., 2011a; Shi et al., 2011b; Lin et al., 2012).

### Liquid diet treatment

To minimize fecal accumulation and release lumen distention in inflammation, some rats were fed exclusively with *Ensure* liquid diet (Abbott Nutrition, Lake Forest, IL) *ad libitum* (Lin et al., 2021) after intracolonic instillation of saline or TNBS. The average daily intake of the liquid diet for a control rat is about 75 mL with 75 kcal (3.0 g of protein, 2.0 g of fat, 11 g of carbohydrate and 0.3 g of fiber). Rats were housed separately in wire-bottomed cages to prevent them from eating feces or bedding materials (Lin et al., 2021). All rats had continuous free access to water.

In our pilot study testing the effect of different liquid diets, we tried not only *Ensure* liquid diet but also a rodent liquid diet, #D11112201L made by Research Diets (New Brunswick, NJ). Although the rodent liquid diet is not identical as *Ensure* liquid diet in terms of nutrient contents, it had very similar effect as *Ensure* liquid diet in eliminating mechanical distention in the colon. We decided to use *Ensure* liquid diet in the study, as it has been used in the EEN treatment for CD patients and in eliminating mechanical distention in rats (Lin et al., 2021). Given that liquid diet may affect microbiota and microbial metabolites (Narula et al., 2018; Ashton et al., 2019), and nutrient contents, caloric density, food osmolarity, and fibers may affect motility, we included both control and TNBS rats in each diet group (regular pellet food or liquid diet).

### Tissue collection

**FIGURE 1:**
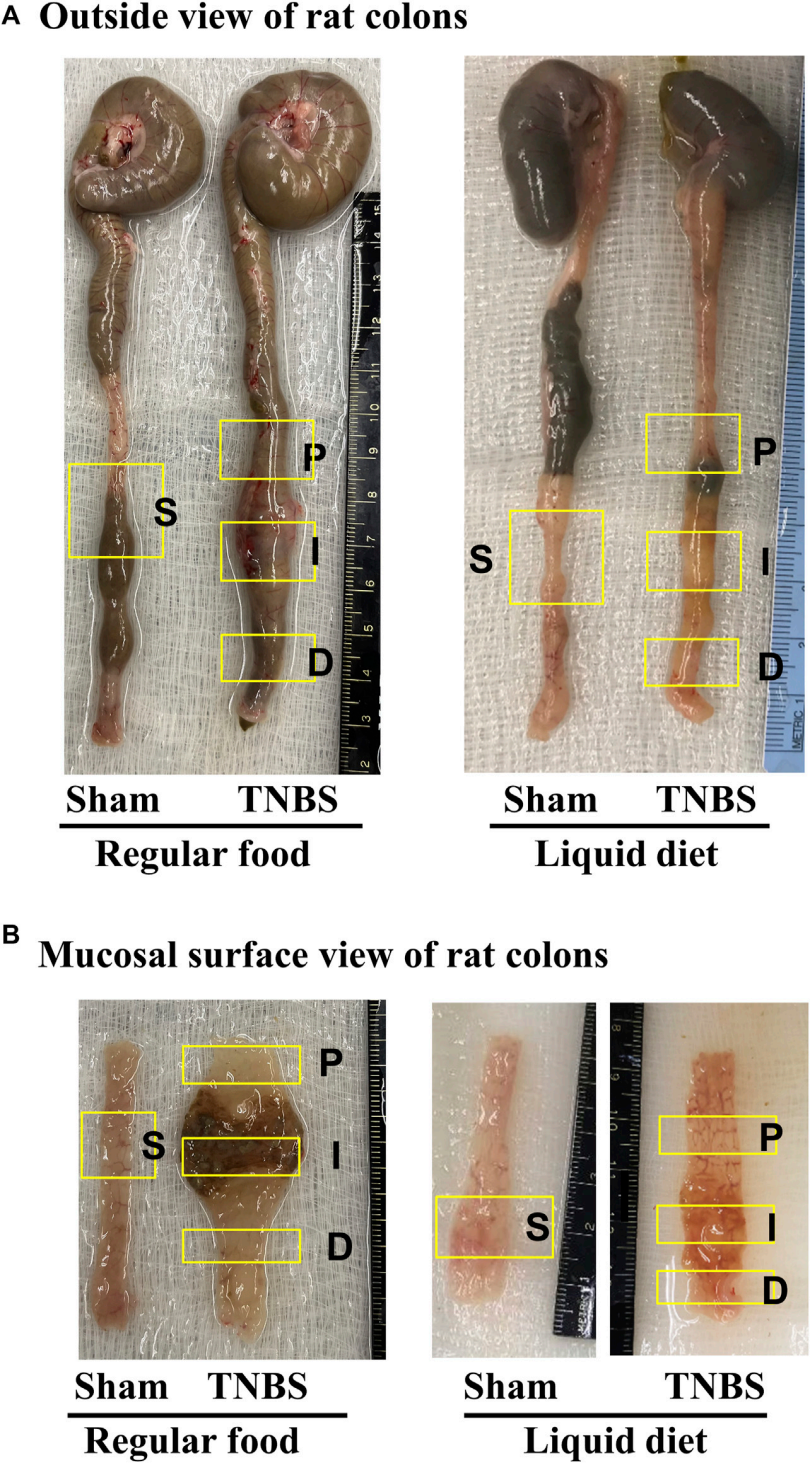
Rat model of TNBS-induced Crohn’s-like colitis: effect of liquid diet treatment. **(A)** Outside view of rat colons and **(B)** mucosal surface view of distal colons. Rat model of Crohn’s-like colitis was induced by intracolonic instillation of TNBS (65 mg/kg in 250 μL of 40% ethanol) to the distal colon. When rats were fed with regular pellet food, TNBS treatment induced localized transmural inflammation (∼2 cm length) in the distal colon (site I), with a distended colon segment (site P) prior to the inflammation site and a non-distended segment (site D) distal to the inflammation site. However, when rats were fed exclusively with liquid diet (mimicking exclusive enteral nutrition), lumen distention in site P was eliminated.

Immediately after rats were euthanized, the colon was isolated, and collected in fresh and carbogenated Krebs buffer (in mmol/l: 118 NaCl, 4.7 KCl, 2.5 CaCl2, 1 NaH2PO4, 1.2 Mgcl2, 11 days-glucose, and 25 NaHCO3). After cleansing, the colon was opened along the mesenteric border, and pinned flat in a Petri dish with Sylgard base (Shi and Sarna, 2004; Shi et al., 2011b; Geesala et al., 2022). Different colonic sites (site I, site P, and site D) of TNBS treated rats were identified as shown in the figure (Figure 1), and colonic tissue of 1–2 cm-long was taken from each site for molecular and functional studies. The colonic site of inflammation where TNBS was instilled to (typically 5–7 cm from the anus) was identified as site I. The colonic portion 2–3 cm proximal to the oral margin of inflammation was identified as site P. The colonic portion 1–2 cm distal to the aboral margin of inflammation was considered site D. For saline treated control rats, the counterpart colon tissues were taken accordingly. The full-thickness tissue, mucosa/submucosa, and muscularis externa were prepared from each site as described previously (Shi and Sarna, 2004; Shi et al., 2011b; Geesala et al., 2022). The muscularis externa tissues were processed immediately for muscle bath experiments. Other tissue samples were frozen in liquid nitrogen and stored at −80°C for molecular studies. Some full thickness samples were saved in 10% formaldehyde for immunohistochemistry study.

### Immunohistochemistry study

Immunohistochemistry staining of COX-2 was performed as described previously (Shi et al., 2011a), on formalin-fixed, paraffin-embedded colon segments of sham control and rats with stenotic CD-like colitis. Sections of 4-μm thickness were blocked with 5% normal goat serum in PBS for 20 min at room temperature, and incubated with the rabbit anti-COX-2 antibody (1:200, Cayman Chemical) and a biotin-conjugated anti-rabbit secondary antibody (Vector Laboratories, Burlingame, CA). After being incubated with avidin-biotin complex (Vector kit, Vector Laboratories), the sections were stained in diaminobenzidine tetrahydrochloride with 0.03% hydrogen peroxide. As a negative control, sections of the same specimens were processed by the same method but omitting the anti-COX-2 primary antibody.

### RNA extraction and quantitative RT-PCR

Total RNA was extracted from tissues using the Qiagen RNeasy kit (Qiagen, Valencia, CA). One microgram of total RNA was reverse transcribed by using the SuperScript III First-Strand Synthesis System (Invitrogen, Carlsbad, CA) (Shi et al., 2007; Shi and Sarna, 2008; Shi et al., 2011b). Real-time quantitative RT-PCR was performed using the Bio-Rad CFX96 Real-Time PCR system (Hercules, CA), as described previously (Lin et al., 2021; Geesala et al., 2022). The TaqMan probe for detection of rat COX-2 (Rn00568225-m1) was purchased from Invitrogen. The fold-change relative to control was calculated with the comparative C_T_ (ΔΔCT) method with endogenous reference 18S rRNA (Part no. 4352930E, Applied Biosystems) as the normalizer.

### Enzyme immunoassay of PGE_2_

Rat colonic muscularis externa tissue was homogenized in cold PBS (in mmol/l 137 NaCl, 2.7 KCl, 10 Na2HPO4, KH2PO4, pH 7.4) supplemented with protease inhibitors for protein extraction. PGE_2_ was measured with the PGE_2_ enzyme immunoassay kit from Cayman Chemical (Ann Arbor, MI) by following the manufacturer’s protocols (Shi et al., 2011a; Lin et al., 2012; Lin et al., 2021).

### Muscle bath experiments

Freshly obtained colon segments were opened along the mesenteric border, cleansed, and pinned flat in a Petri dish with Sylgard base in carbogenated Krebs solution. The muscularis externa layer was collected for muscle bath experiments. The smooth muscle strips (3 mm × 10 mm) were mounted along the circular muscle orientation in individual muscle baths (Radnoti Glass, Monrovia, CA) filled with 10 mL of carbogenated Krebs solution at 37°C. The contractile activity was recorded as previously described (Shi and Sarna, 2008; Lin et al., 2012; Hegde et al., 2022) with Grass isometric force transducers and amplifiers connected to Biopac data-acquisition system (Biopac Systems, Goleta, CA). The muscle strips were equilibrated in the muscle bath under 1 g tension for 60 min at 37°C before they were tested for contractile response to cholinergic activation. In the last 10 min of the equilibration period, we assessed the spontaneous contractile activity of the muscle strips by taking the average of two 2-min measurements of area under contractions (AUC) with one measurement at the time with highest contractile activity and another with lowest contractile activity of the strips. The smooth muscle contractility in response to cholinergic activation was tested by obtaining concentration-response curves to acetylcholine (ACh 10^–6^ to 10^–2^ M) in the muscle bath. ACh doses were tested one by one incrementally with the lowest dose first and the highest dose last with an interval of 2–3 min between each dose of ACh. The ACh-evoked contractile response was quantified as the increase of AUC during 2 min after addition of each ACh dose to the bath over the baseline AUC during 2 min before the addition of the lowest dose of ACh. The tissue dry weight of each muscle strip was used to normalize the contractile activities.

### Statistical analysis

All data points are expressed as means ± SEM. Statistical analysis was performed by analysis of variance with non-repeated measures (by Student-Newman-Keuls test) for comparisons of multiple groups and Student’s t-test for comparisons of two groups. A *p*-value of ≤0.05 was considered statistically significant.

## Results

### Site-specific changes of colonic smooth muscle contractility in Crohn’s-like colitis.

When rats were fed with regular pellet food, intracolonic instillation of TNBS led to localized transmural inflammation with stenosis in the instillation site (site I) and lumen distention in the site proximal to inflammation (site P). Though mechanically distended, site P did not show visible inflammation. Interestingly, the colonic site distal to inflammation (Site D) showed neither distention nor inflammation (Figures 1A,B). However, when rats were fed exclusively with liquid diet, mechanical distention in site P was eliminated in the TNBS induced colitis rats (Figures 1A,B, right panel). Images are representative of 5 rats in each group.

**FIGURE 2:**
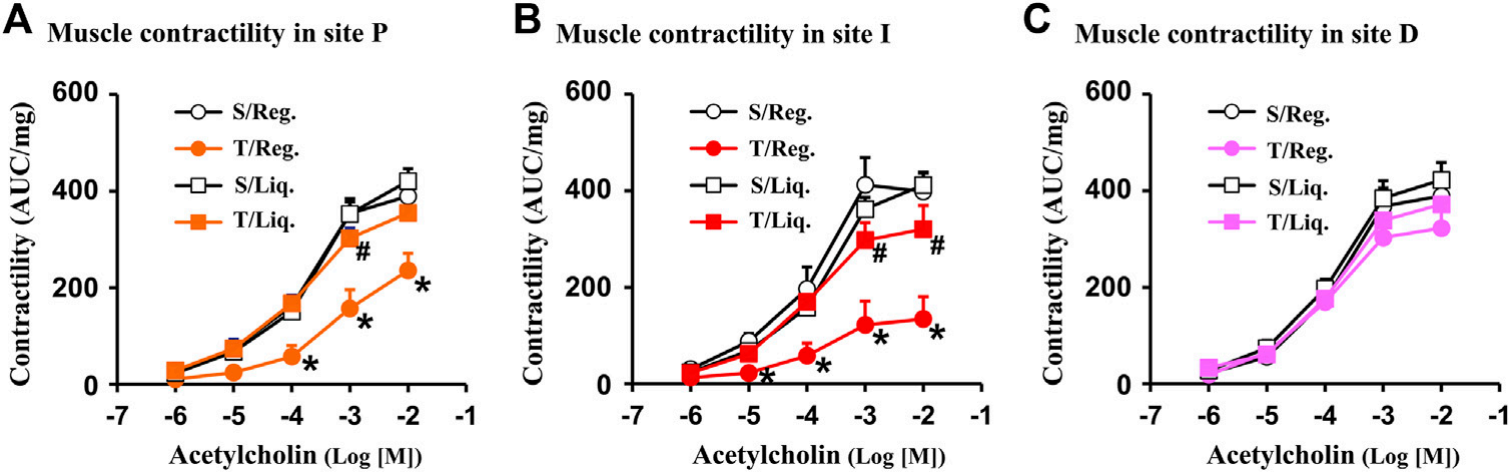
Smooth muscle contractility in Crohn’s-like colitis. Shown are results of circular muscle contractility in site P **(A)**, site I **(B)** and site D **(C)** of TNBS colitis (day 7). Note: contractility was significantly suppressed in site P and I, but not in site D of CD rats (7 days). N = 5. *p < 0.05 vs. sham. S, sham; T, TNBS; Reg, regular pellet food; Liq, liquid diet (EEN).

With the CD-like colitis model, we first determined whether colonic smooth muscle contractility was altered at the three distinct sites (I-site, P-site, D-site) in the colitis rats compared to sham controls when they were fed with regular solid food (Figures 2A–C). We found that colonic circular muscle contractile response to cholinergic agent acetylcholine (ACh 10^−6^ ∼ 10^−2^ M)) was dramatically suppressed in the I-site compared to the corresponding site of control rats (*p* < 0.05 vs. sham control. N = 5 each group) (Figure 2B). This result is consistent with previous reports (Shi et al., 2011b). More importantly, the muscle contractility was significantly suppressed also in the P-site, which is mechanically distended but not with obvious inflammation (Figure 2A). However, the muscle contractility did not show any significant suppression in the D-site, which presents neither inflammation nor distention (Figure 2C). The site-specific changes of smooth muscle contractility suggest that mechanical stress, rather than inflammation itself, may be an independent pathogenic factor contributing to muscle dysfunction in Crohn’s-like colitis.

### Effect of liquid diet treatment on site-specific changes of colonic smooth muscle contractility in Crohn’s-like colitis

Liquid diet treatment was found to release fecal retention in constipation (Lin et al., 2021). In the present study, we found that liquid diet treatment also eliminated lumen distention in the CD-like colitis model in which inflammatory stenosis would lead to massive fecal retention and lumen distention if rats were fed with solid food (Figure 1).

We then determined the site-specific changes of muscle contractility in colitis when rats were fed exclusively with liquid diet and mechanical distention was eliminated in the colitis rats. Our results showed that muscle contractility was significantly improved in the I-site and largely normal in the P-site in inflammation when the rats were fed with liquid diet (Figures 2A,B). Interestingly, at the non-distended D-site of the colon, there was no significant change in SMC contractility in inflammation in rats fed either with regular solid diet or liquid diet (Figure 2C).

### Site specific changes of COX-2 expression in Crohn’s-like colitis rats fed with regular solid diet and liquid diet

**FIGURE 3:**
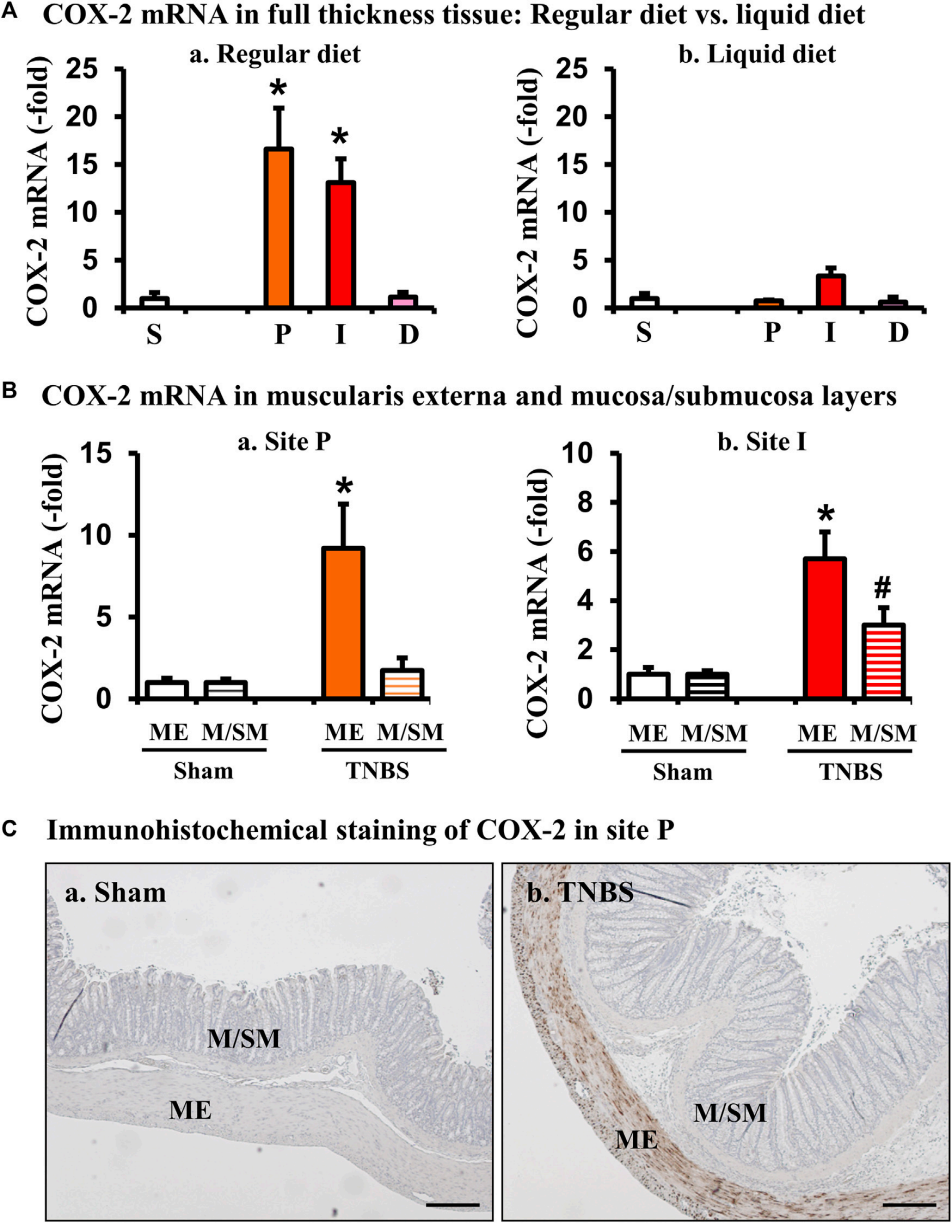
Site- and tissue-specific expression of COX-2 in the colon in Crohn’s-like colitis. **(A)** COX-2 mRNA expression in full-thickness colon tissues of sham and colitis in sites P, I, and D. COX-2 mRNA expression was markedly induced in sites I and P, but not D in TNBS rats (7 days) when fed with regular diet (a). However, liquid diet treatment blocked upregulation of COX-2 (b). N = 4 or 5. *p < 0.05 vs. S (sham). **(B)** COX-2 mRNA expression in the muscularis externae (ME) and mucosa/submucosa (M/SM) in site P (a) and site I (b). Colon tissue from site P of sham and TNBS colitis rats (day 7) was separated into muscularis externae (ME) and mucosa/submucosa (M/SM). Expression of COX-2 mRNA was quantitated by qRT-PCR. N = 4 in each group. *p < 0.05 vs. sham ME. #*p* < 0.05 vs. sham M/SM. **(C)** Tissue source of COX-2 protein expression (brown) in site P were further confirmed by immunohistochemistry. Scale bars = 100 µm.

COX-2, via COX-derived prostaglandins, has profound effects on smooth muscle function in the gut (Li et al., 2012). Our previous studies demonstrated that expression of COX-2 is highly sensitive to mechanical stress in the gut, as circumferential mechanical stretch (i.e., lumen distention) leads to mechano-sensitive expression of COX-2 exclusively in gut smooth muscle cells in a model of mechanical bowel obstruction (Shi et al., 2011a; Shi, 2017). To determine if COX-2 expression is also induced in stenosis-associated distention in the colitis model, we then determined the site-specific expression of COX-2 mRNA in sites P, I, and D in the Crohn’s-like colitis model. When rats were fed with regular solid food, COX-2 mRNA expression increased 13.1 (±2.5)-fold in the I-site of colitis rats, compared to sham controls (*p* < 0.05 vs. sham control. N = 4 or 5) (Fig. 3Aa). Furthermore, COX-2 mRNA expression was increased significantly also in the mechanically distended P-site [16.6 (±4.3)-fold] (Figure 3A). However, when rats were given a 7-day regimen of exclusive liquid diet to eliminate mechanical stress in the model, the level of COX-2 mRNA expression was dramatically attenuated in the inflamed I-site and the distended P-site (N = 5) (Fig. 3Ab). Importantly, expression of COX-2 mRNA was not significantly changed in the non-distended D-site in the CD-like colitis rats even if the rats were fed with solid food (Figure 3A). This data indicates that expression of COX-2 in the P-site is largely dependent on mechanical stress. Lastly, we wanted to determine where COX-2 expression occurred within the colonic tissue layers in the P-site, and found that increased COX-2 expression occurred mainly in the muscularis externae (ME) layer of the distended colon segment, particularly in the colonic smooth muscle cells (N = 4) (Figures 3B,C).

### Site specific changes of PGE_2_ production in Crohn’s-like colitis rats fed with regular solid food and liquid diet

**FIGURE 4:**
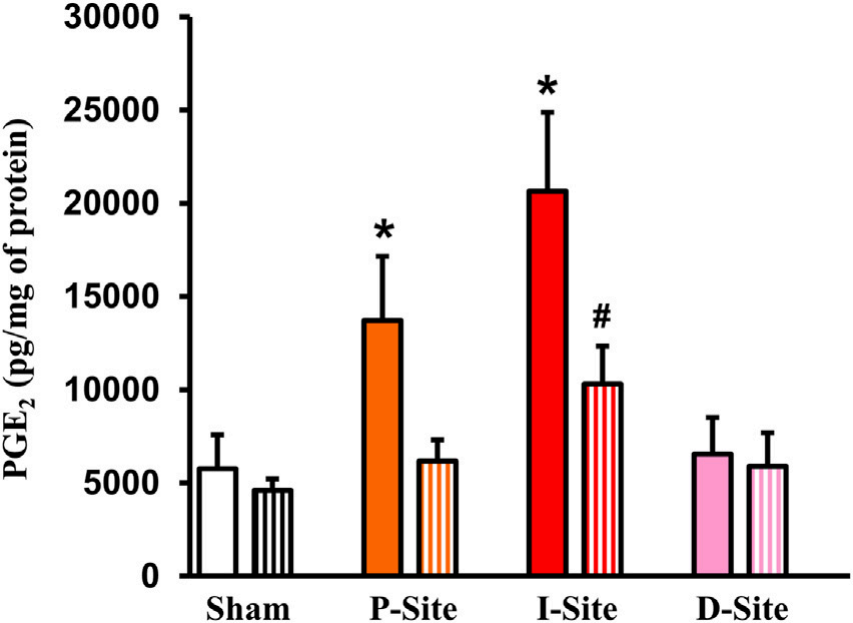
PGE_2_ levels in sham colon and different colonic sites of Crohn’s-like colitis. Full-thickness colon tissues were taken for the measurements of PGE_2_. Shown are PGE_2_ levels in sham and different sites (P, I, and D) of TNBS colitis (day 7) with regular diet (solid bar) and liquid diet treatment (streaked bar). Note: PGE_2_ levels were significantly increased in site P and I, but not in site D of CD-like rats. Liquid diet treatment blocked PGE_2_ increase in the P-site. N = 5 or 6. *p < 0.05 vs. sham. #*p* < 0.05 vs. I-site/regular food.

PGE_2_ is among the most potent prostaglandins derived from COX-2 and found to suppress colonic smooth muscle contractility in rats (Lin et al., 2012). We next determined PGE_2_ levels in different sites of the colonic tissues in sham and colitis rats treated with solid food and liquid diet. When rats were fed with solid food, PGE_2_ levels were increased significantly not only in the I-site (20,653 ± 4,233 pg/μg of protein, *p* < 0.01. N = 5 all groups) but also in the P-site (13,708 ± 3,450 pg/μg, *p* = 0.01. N = 5) of the colitis colon, compared to sham colon (5,776 ± 1,813 pg/μg) (Figure 4). However, the D-site (6,555 ± 1958 pg/μg) did not exhibit any significant increase of PGE_2_. Interestingly, liquid diet treatment blocked the PGE_2_ increase in the P-site and significantly suppressed PGE_2_ level in the I-site (10,306 ± 2,046 pg/μg, *p* < 0.01 vs. the I-site level in solid food-treated rats) (Figure 4).

### Effect of COX-2 inhibitor NS-398 on colonic smooth muscle cell contractility

**FIGURE 5:**
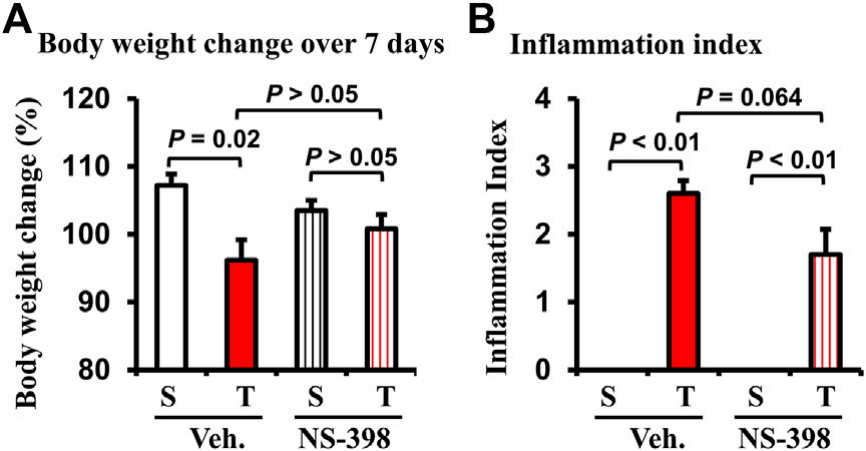
Effect of NS-398 treatment on body weight change and inflammation index in Crohn’s-like colitis rats. NS-398 (5 mg/kg, i. p. daily) and vehicle control (DMSO saline) were administered daily to sham and TNBS-induced Crohn’s-like colitis rats. The percentage changes of body weight over 7 days of treatment (after TNBS instillation and vehicle or NS-398 treatment) were determined **(A)**. The inflammation index (1–3) was scored **(B)** on the day when animals were euthanized, and the colon was taken out. N = 4 or 5 rats. *p* values are given for the comparisons. S, sham; T, TNBS treated rats (Crohn’s-like colitis); Veh, vehicle.

Lastly, we determined if COX-2 plays a role in smooth muscle dysfunction in the CD-like colitis model. COX-2 inhibitor NS-398 (5 mg/kg, i. p. daily) or vehicle control was administered in sham and colitis rats daily. Rats were fed with regular pellet food and euthanized 7 days later. The body weight was decreased significantly over the 7-day course in TNBS-induced colitis rats when treated with vehicle (Figure 5A *P* = 0.02 vs. sham. N = 4 or 5 rats in each group). However, when rats were administered with NS-398, TNBS colitis rats did not demonstrate any significant body weight decrease compared to sham control rats (Figure 5A). NS-398 administration did not significantly affect body weight change in sham control rats (*p* > 0.05 between sham/vehicle and sham/NS-398). We also assessed inflammation in the rats. NS-398 showed a trend to reduce inflammation in TNBS colitis rats (*p* = 0.064 vs. TNBS colitis with vehicle administration. N = 4 or 5) (Figure 5B). However, even with NS-398 administration, the colon inflammation in the TNBS colitis rats was still obvious (*p* < 0.01 vs. sham control.) (Figure 5B).

**FIGURE 6:**
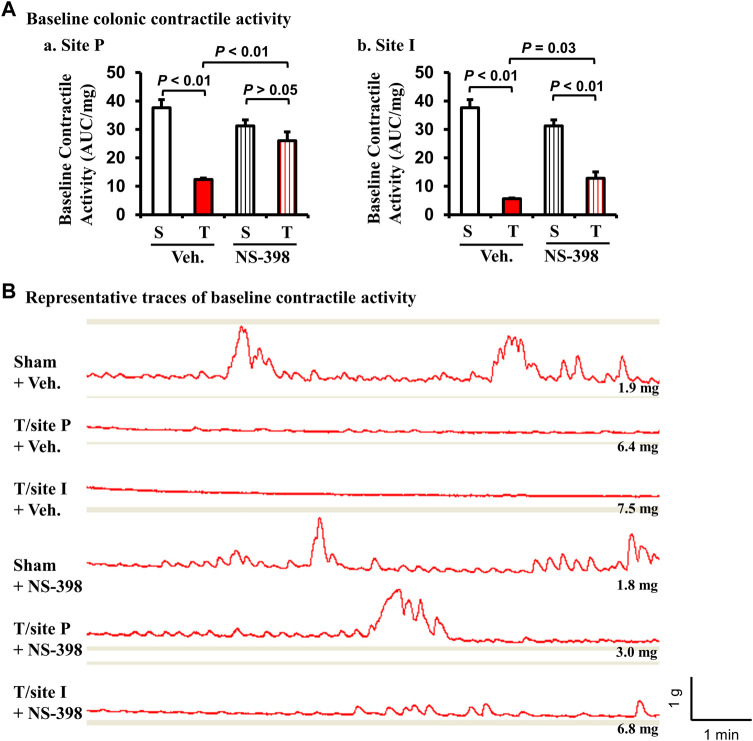
Effect of NS-398 on baseline colonic smooth muscle contractile activity in Crohn’s-like colitis rats. NS-398 (5 mg/kg, i. p. daily) and vehicle control (DMSO saline) were administered daily to sham and TNBS-induced Crohn’s-like colitis rats. Colonic smooth muscle strips were prepared and mounted at 1 g of tension for 40–50 min in tissue bath. The integral contractile activity (area under contractions, AUC) was determined with the average of two measurements of activity for 2 min. With vehicle treatment, TNBS colitis rats demonstrated a significantly lower baseline colonic contractile activity than in sham rats at both P and I sites (A-a and A-b, respectively). NS-398 treatment significantly improved colonic baseline activity in TNBS colitis rats at both P-site and I-site **(A–a, A–b)**. However, even with NS-398 treatment, the baseline activity was still reduced in TNBS rats compared to sham control at the I-site **(A,B)**. Representative traces for all groups are shown in **(B)**. The number at the right bottom of each trace is the tissue weight of the muscle strip. The vertical and horizontal bars of the insert at the right bottom of panel B indicate 1 g of tension and 1 min of time, respectively for all traces. N = 5 rats. *p* values are given for the comparisons in **(A)**. S, sham; T, TNBS treated rats (Crohn’s-like colitis); Veh, vehicle.

The baseline contractile activity of colonic muscle strips was significantly decreased in TNBS colitis rats in both P-site and I-site in the DMSO vehicle treatment group (*p* < 0.01 vs. sham control. N = 5 each group) (Fig. 6Aa and Ab). However, with NS-398 administration, the baseline contractile activity in the P-site of TNBS colitis rats was not different from sham control (*p* > 0.05 vs. sham control. N = 5) (Fig. Aa). The baseline activity of the I-site was still significantly reduced compared to sham control (*p* < 0.01) even with NS-398 treatment. However, NS-398 treatment improved contractile activity in the I-site compared to the same site with vehicle treatment (*p* = 0.03) (Fig. 6Ab). Representative traces of colonic baseline contractile activity were shown for each group (Figure 6B). NS-398 administration did not significantly affect colonic baseline activity in sham control rats (*p* > 0.05 between sham/vehicle and sham/NS-398).

**FIGURE 7:**
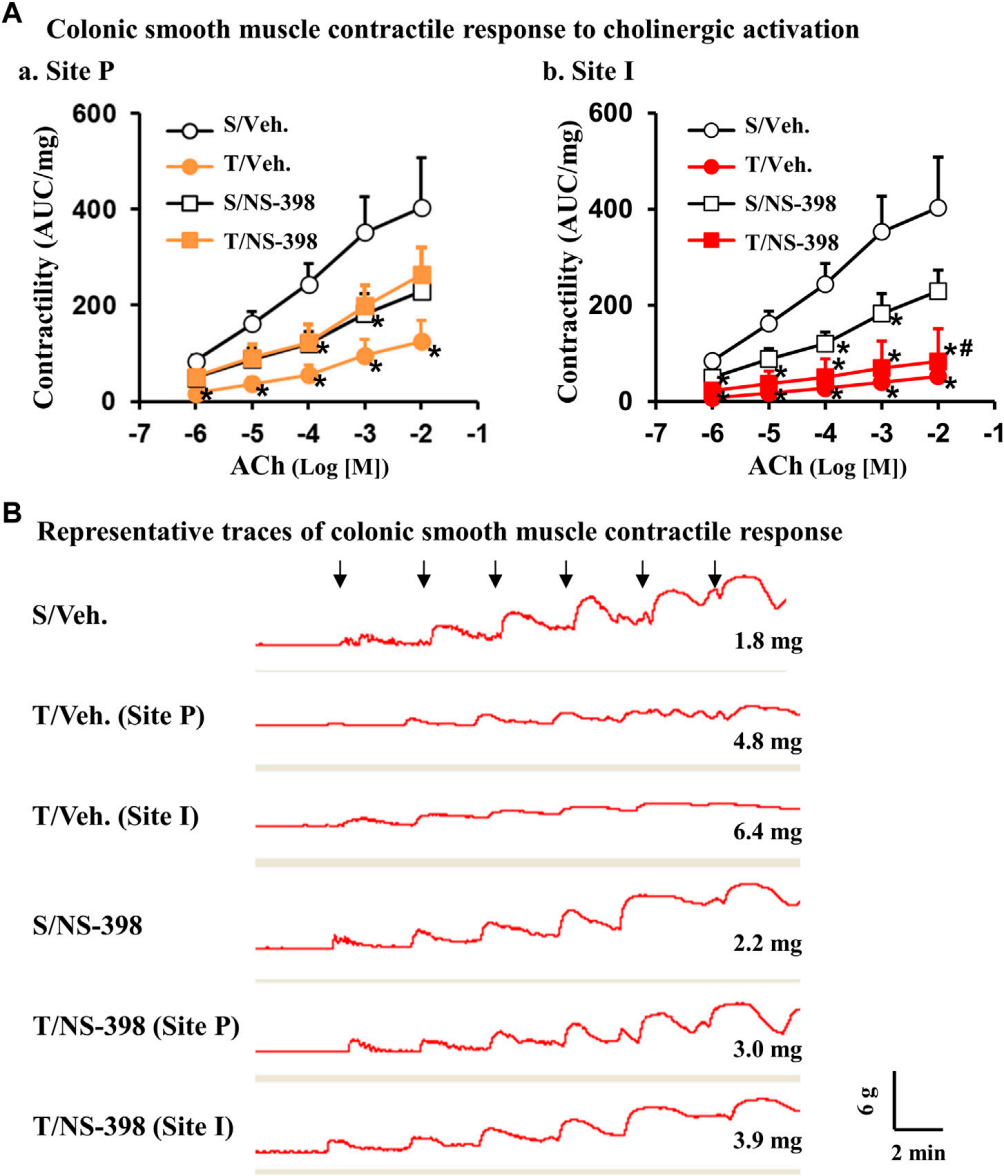
Effect of COX-2 inhibitor NS-398 on smooth muscle contractile response to cholinergic activation in Crohn’s-like colitis rats. NS-398 (5 mg/kg, i. p. daily) and vehicle control (DMSO saline) were administered daily to sham and TNBS-induced Crohn’s-like colitis rats. With vehicle treatment, TNBS colitis rats demonstrated a significantly lower colonic smooth muscle contractility than in sham rats in response to all ACh concentrations at both P and I sites **(A,a)** and **(A,b)**, respectively. With NS-398 treatment, colonic smooth muscle contractility in TNBS colitis rats was not significantly different from sham at the P-site for all ACh concentrations **(A–A)**. Smooth muscle contractility at the I-site was not significantly different between sham and TNBS colitis rats for all ACh concentrations, except that of ACh 10^−2^ M **(A,B)**. Representative traces for all groups are shown in **(B)**. The signs of “↓” indicate the addition of ACh 10^−7^ M, 10^−6^ M, 10^−5^ M, 10^−4^ M, 10^−3^ M, and 10^−2^ M, respectively from left to right. The number at the right bottom of each trace is the tissue weight of the muscle strip. The vertical and horizontal bars of the insert at the right bottom of panel B indicate 6 g of tension and 2 min of time, respectively for all traces. N = 4 or 5 rats. *p < 0.05 vs. sham/veh. #*p* < 0.05 vs. sham/NS-398. S, sham; T, TNBS treated rats (Crohn’s-like colitis); Veh, vehicle.

We also studied muscle contractile response to cholinergic agonist ACh and found that in the TNBS-treated colitis rats given a DMSO vehicle control, there was a significant reduction of muscle contractility in response to all ACh concentrations (10^–6^ to 10^–2^ M) in both the I-site and the P-site (*p* < 0.05, N = 4 or 5) (Figure 7 Aa and Ab). Interestingly, when TNBS-treated rats were given an i. p administration of NS-398, there was no significant difference (*p* < 0.05, N = 4 or 5 rats) in muscle contractility at the distended P-site for all ACh concentrations, indicating that COX-2 may play a critical role in smooth muscle dysfunction in the pre-inflammation site (Fig. 7Aa). NS-398 treatment also partially improved muscle function in the inflammation site (I-site), as smooth muscle contractility at the I-site was not significantly different between sham and TNBS colitis rats for all ACh concentrations, except that of ACh 10^−2^ M (*p* < 0.05, N = 4 or 5 rats) (Fig. 7Ab). Compared to vehicle treatment, NS-398 administration decreased the contractile response of control colons to ACh 10^−4^ and 10^−3^ (M) (Fig. 7Aa and Ab). Representative traces of smooth muscle contractile response to ACh doses were shown for each group (Figure 7B). We confirmed that NS-398 treatment effectively blocked PGE_2_ production at P-site (6,328 ± 1,452 pg/μg vs. 3,120 ± 1,150 pg/μg in sham with NS-398, *p* = 0.10) and significantly decreased PGE_2_ level at I-site (9,260 ± 1,610 pg/μg) compared to the I-site level in rats with no NS-398 treatment (20,653 ± 4,233 pg/μg, *p* < 0.05. N = 5).

## Discussion

Gastrointestinal symptoms such as diarrhea, constipation, abdominal distention, and pain, are well documented in IBD patients (Snape et al., 1991; Camilleri, 2011; Bickelhaupt et al., 2013; Menys et al., 2016). Gut smooth muscle dysfunctions, i.e., suppressed contractility, may account for the altered motility, abdominal distention, and cramping in IBD (Sarna et al., 2006; Lomax et al., 2017). Based on the studies on the inflamed tissues in animal models and patients with IBD, several cytokines and inflammatory mediators are proposed to play a role in gut smooth muscle dysfunction during inflammation in IBD (Shi et al., 2003; Sarna et al., 2006; Lomax et al., 2017). Immune cell-derived cytokines such as IL-1β, TNF-α and IL-12 were found to contribute to decreased contractility of gut smooth muscle in inflammation (Neurath et al., 1995; Kinoshita et al., 2006; Kiyosue et al., 2006; Ohama et al., 2007). These mediators may act on gut SMCs to suppress L-type Ca^2+^ channel, intracellular signaling molecules, and CPI-17, to decrease myosin light chain (MLC) phosphorylation (Shi et al., 2005; Ohama et al., 2007). However, clinical data suggest that anti-inflammatory treatments, though reducing inflammation, are not very effective in attenuating gastrointestinal symptoms (Snape et al., 1991; Annese et al., 1997). In fact, gastrointestinal symptoms such as diarrhea, constipation, abdominal distention, and pain may be present in IBD patients when they do not have active inflammation (Snape et al., 1991; Zaman et al., 2002; Camilleri, 2011; Bickelhaupt et al., 2013). Recent studies found that motor activity is suppressed not only in the inflammation site, but also in the unaffected bowel segment prior to the site of inflammation in CD (Bickelhaupt et al., 2013; Menys et al., 2016). These studies also provide direct evidence that aberrant motility (i.e., reduced motility variance) in morphologically normal bowel prior to the site of inflammation is associated with patient symptoms and inflammation burden in CD (Bickelhaupt et al., 2013; Menys et al., 2016). Mechanisms behind the aberrant motility in the inflammation site and particularly the bowel segment prior to inflammation are not clear.

We found in the present study that mechanical stress may act as a non-inflammatory factor in the pathogenesis of smooth muscle dysfunction in CD, at least in the non-inflamed segment prior to inflammation. As previously shown (Shi et al., 2011b; Geesala et al., 2022), the rat model of TNBS-induced colitis mimics well stenotic Crohn’s disease, as intracolonic instillation of TNBS induced inflammation-associated stenosis at the site of instillation (I-site) and marked mechanical distention in the site prior to the stenosis (P-site). Although mechanically distended, the P-site does not demonstrate apparent inflammation. However, smooth muscle contractility is suppressed, and COX-2 expression is increased in the P-site as in the I-site, but not in the segment distal to stenosis. These results strongly suggest that mechanical stress may be a pathogenic factor in smooth muscle dysfunction. We also found that COX-2 expression and PGE_2_ production are increased especially in the smooth muscle tissue in the P-site, but not in the distal site, indicating that COX-2 expression in the P-site is mechanical stress-dependent. Previous studies *in vitro* and *in vivo* demonstrated that COX-2 expression in gut smooth muscle cells is highly responsive to mechanical stress (Li et al., 2012; Lin et al., 2014). Moreover, elimination of mechanical distention with liquid diet treatment blocked COX-2 expression and improved smooth muscle function in the colitis model. This result confirms that mechanical stress is an independent pathogenic factor in the development of smooth muscle dysfunction in the colitis model. Finally, administration of COX-2 inhibitor almost completely restored colonic smooth muscle function (both spontaneous contractile activity and ACh-evoked contractile response) in the P-site of the CD-like colitis rats. These results suggest that mechanical stress-induced expression of COX-2 and production of PGE_2_ contribute to smooth muscle dysfunction, especially in the P-site. Previous study found that EP2 and EP4 receptors are responsible for COX-2 and PGE_2_ mediated suppression of smooth muscle contractility in a mechanical obstruction model (Lin et al., 2012). However, further research is warranted to better understand the mechanisms of COX-2-derived prostaglandins on gut smooth muscle and neuromuscular control of gut motility in CD.

Our results showed that inhibition of COX-2 activity with NS-398 administration not only improved colonic smooth muscle function, but also blocked body weight decreases in colitis rats. Interestingly, NS-398 treatment did not significantly reduce inflammation. This data indicates that improved gut smooth muscle function and motility during gut inflammation may contribute to the wellbeing of the animals with colitis. We noticed that compared to vehicle treatment, NS-398 administration did not affect baseline contractile activity of the control colon but decreased the contractile response to ACh 10^−4^ and 10^−3^ (M). We do not know exactly what accounts for these changes. However, although NS-398 is considered a specific COX-2 inhibitor, its possible effects on COX-1 or other non-specific targets and associated changes of various prostaglandins may have complicated effect on smooth muscle contractions especially high-amplitude contractions as that induced by high doses of ACh. Nevertheless, we used the same doses of NS-398 (5 mg/kg) and vehicle control (DMSO) for sham and colitis rats to minimize any non-specific effect of NS-398 on muscle contractility.

It is noteworthy that mechanical stress is commonly encountered in gut inflammation. Inflammatory infiltration, edema, tissue deformation and fibrosis in the inflamed tissue (Shi, 2017; Geesala et al., 2021) are all considered to present mechanical stress to the tissue involved. The I-site, with inflammation, stenosis, and distention in the CD-like colitis model, is affected not only by inflammation but also by mechanical stress. We found that COX-2 mRNA expression is upregulated in the I-site as well as P-site in the full-thickness tissues in colitis. However, the increase of COX-2 occurred in both mucosa/submucosal layer and muscularis externa in the I-site, but only in the muscularis externa in the P-site. These data suggest that increased COX-2 in the I-site may be due to both inflammation and mechanical stress. Because it is almost impossible to distinguish mechanical stress from inflammation in the I-site *in vivo*, we focused our effort on the pre-inflammation site in the present study. Our study suggests that mechanical stress, via mechano-transcription of mechanosensitive molecules such as COX-2, may play a crucial role in smooth muscle dysfunction in CD.

The current goal of treatment in CD is to induce prolonged remission. Corticosteroids, immune modulators, and biologic agents are all found to be effective in reducing inflammation (Sidoroff and Kolho, 2012; Cheifetz, 2013). Unfortunately, most of these treatment options have substantial adverse effects and may lose efficacy over time. Corticosteroids are limited in their use by risks of infection, osteoporosis, growth retardation, poor mucosal healing, and early relapses on cessation of therapy (Sidoroff and Kolho, 2012; Forbes et al., 2017). This is especially problematic in pediatric patients who may experience significant growth retardation and osteoporosis with steroid therapy (Forbes et al., 2017). Thus, safe, and effective therapies, especially diet-based treatments are much appreciated by CD patients (Cheifetz, 2013; Ashton et al., 2019). Exclusive enteral nutrition (EEN) is the only established dietary treatment for CD (Narula et al., 2018; Ashton et al., 2019). It involves oral or nasogastric tube feeding of a complete liquid diet with exclusion of normal foods for a defined period (usually 4–8 weeks). Over the last 2 decades, EEN has emerged as a highly effective treatment for the induction of remission in CD. It is low-risk, steroid-sparing, and now the first-line therapy in children with reported remission rates up to 80% (Narula et al., 2018; Ashton et al., 2019). There is evidence that EEN is also effective in adult CD patients (Mowat et al., 2011; Yang et al., 2017). However, the evidence for the efficacy of EEN in adult patients is weaker than in pediatric population, possibly due to practicalities of use (compliance, tolerability, etc.) (Mowat et al., 2011; Ashton et al., 2019). EEN treatment not only reduces inflammation but also significantly improves patients’ disease activity and bowel symptoms (Hyams et al., 1991; Thia et al., 2011; Ashton et al., 2019). However, the exact mechanisms for the therapeutic benefits of EEN on inflammation and gut function in CD are still not known. In our study, exclusive liquid diet was applied to animals to eliminate lumen distention due to fecal retention. This treatment mimics exactly EEN (Lin et al., 2021). We found that EEN treatment with the liquid diet restores colonic smooth muscle function in CD-like colitis via eliminating mechanical stress-induced COX-2. EEN treatment also significantly reduced inflammation and decreased expression of pro-inflammatory mediators in the model. Together, our studies suggest a novel mechanism of action of EEN in CD that EEN eliminates mechanical stress and mechano-transcription of pro-inflammatory mediators and COX-2 to reduce inflammation and improve bowel symptoms. It is interesting to note that ulcerative colitis, a type of IBD with superficial inflammation and no stricture formation, is not as responsive to EEN treatment as Crohn’s disease (Turner et al., 2012; Ashton et al., 2019). This may further imply that the mechanism of EEN to reduce inflammation and improve bowel function in CD may be because it decreases mechanical stress in the gut.

Liquid diet, such as EEN for the management of Crohn’s disease, may affect gut microbiota composition and diversity (MacLellan et al., 2017; Ashton et al., 2019). It was postulated that the effect of EEN treatment on gut microbiota may be responsible for its benefits in CD (Narula et al., 2018; Ashton et al., 2019). However, comprehensive studies and analyses showed that these microbiota changes do not account for the benefits of EEN for Crohn’s. In fact, it was found that EEN treatment reduced gut microbiota diversity (MacLellan et al., 2017; Ashton et al., 2019). To minimize any effect of possible microbiota changes, we included control and TNBS rats in each diet group (normal pellet food or liquid diet). Liquid diet treatment left little or no residual feces in the colon in both control and TNBS rats, indicating the effect of microbiota changes, if any, would be similar in these rats.

Taken together, we found in the stenotic Crohn’s-like colitis model that colonic smooth muscle contractility is suppressed, and COX-2 expression and PGE_2_ production are increased not only in the inflammation site, but also in the distended pre-inflammation site. Liquid diet treatment mimicking EEN regimen released lumen distention, eliminated COX-2 expression and PGE_2_ production, and improved smooth muscle contractility. Administration with COX-2 inhibitor NS-398 significantly improved colonic smooth muscle contractility in the pre-inflammation and inflammation sites in colitis rats. These results suggest that mechano-sensitive expression of COX-2 plays a critical role in colonic smooth muscle dysfunction in Crohn’s-like colitis rats. Our study indicates that the benefits of EEN in CD may depends on its effect to release mechanical stress in the gut.

## Data Availability

The raw data supporting the conclusions of this article will be made available by the authors, without undue reservation.
